# Zanubrutinib Treatment of Central Nervous System Posttransplant Lymphoproliferative Disorder After Allogeneic Hematopoietic Stem Cell Transplantation: A Case Report

**DOI:** 10.3389/fonc.2021.672052

**Published:** 2021-04-29

**Authors:** Ting-Ting Yang, Wei-Hao Chen, Yan-Min Zhao, Hua-Rui Fu, He Huang, Ji-Min Shi

**Affiliations:** ^1^ Bone Marrow Transplantation Center, The First Affiliated Hospital, Zhejiang University School of Medicine, Hangzhou, China; ^2^ Institute of Hematology, Zhejiang University, Hangzhou, China; ^3^ Zhejiang Engineering Laboratory for Stem Cell and Immunotherapy, Hangzhou, China

**Keywords:** hematopoietic stem cell transplantation, EBV-negative, posttransplant lymphoproliferative disorder, lymphoma, zanubrutinib

## Abstract

Posttransplant lymphoproliferative disorder (PTLD) is a rare complication after allogeneic hematopoietic stem cell transplantation (allo-HSCT) with poor prognosis. We report a patient with PTLD involved central nervous system (CNS) who treated with zanubrutinib, a second-generation Bruton tyrosine kinase (BTK) inhibitor. Our report supports the efficacy of bruton tyrosine kinase inhibitor zanubrutinib in the treatment of CNS-PTLD, which provides a new therapeutic option.

## Introduction

Post-transplantation lymphoproliferative disorder (PTLD) is a spectrum of unregulated lymphoid expansion ranging from polyclonal hyperplasia to monoclonal malignant lymphoma, which normally presents with nonspecific signs such as prolonged fever and lymphadenopathy (1). Most PTLDs originate from B cells, associated with Epstein-Barr virus (EBV) reactivation. Compared with PTLD in solid organ transplantation, PTLD after hematopoietic stem-cell transplantation (HSCT) is characteristic of high invasion, early dissemination and high mortality. Currently, there is no consensus regarding the treatment of PTLD.

## Case Presentation

A 39-year-old Chinese man was diagnosed with BCR-ABL-positive acute lymphoblastic leukemia in November 2018. He was treated with 8 cycles of chemotherapy combined with imatinib, resulting in complete remission (CR). He received haploidentical hematopoietic stem cell transplantation (HSCT) from his daughter on September 25, 2019 during the first CR under a myeloablative conditioning regimen (cytarabine, busulfan, cyclophosphamide, methyl-N-2-chloroethyl-N-cyclohexyl-N-nitrosourea, and anti-thymocyte globulin). Prophylaxis against graft-versus-host disease comprised mycophenolate mofetil (MMF), cyclosporine (CsA), and short-course methotrexate (MTX). Neutrophils and platelets were engrafted on days +13 and +14, respectively. Cytogenetic studies showed complete donor chimerism on day +30. The EBV-DNA loads in the blood measured by real-time quantitative polymerase chain reaction were monitored weekly for the first 3 months after transplantation, every 2 weeks from the fourth month posttransplant. Acyclovir was used to prevent virus infection. Once EBV-DNA in the blood was positive, ganciclovir was administered until EBV-DNA turned negative on 2 consecutive measurements.

He was admitted to our center owing to headache, dizziness, and vomiting on day +90. Lumbar puncture revealed that the intracranial pressure exceeded 400 mmH_2_O. Cerebrospinal fluid (CSF) next generation sequencing (NGS) was positive for *Toxoplasma gondii* (*T. gondii*). Toxoplasma serology tests were positive for IgG (16.72 IU/mL), but negative for IgM. Contrast-enhanced brain magnetic resonance imaging (MRI) demonstrated multiple enhancing hyperintense lesions surrounded by edema in bilateral cerebellar and cerebral hemispheres (**Supplementary Figures 1A–C**). He was diagnosed with cerebral toxoplasmosis based on these findings. Oral sulfamethoxazole (SMZ, 1.44 g, thrice daily) and intravenous clindamycin (400 mg, 4 times daily) were administered. His clinical signs resolved completely after 1 week of treatment. After 2 weeks, brain MRI showed a marked decrease in the size of the lesions and perifocal edema. Hence, he was discharged and switched to maintenance therapy with oral azithromycin (500 mg, once daily) and SMZ (0.96 g, twice weekly). Brain MRI performed 1 month after discharge (on day +153 after HSCT) indicated near resolution of the multiple lesions and perifocal edema (**Supplementary Figures 1D–F**).

**Figure 1:**
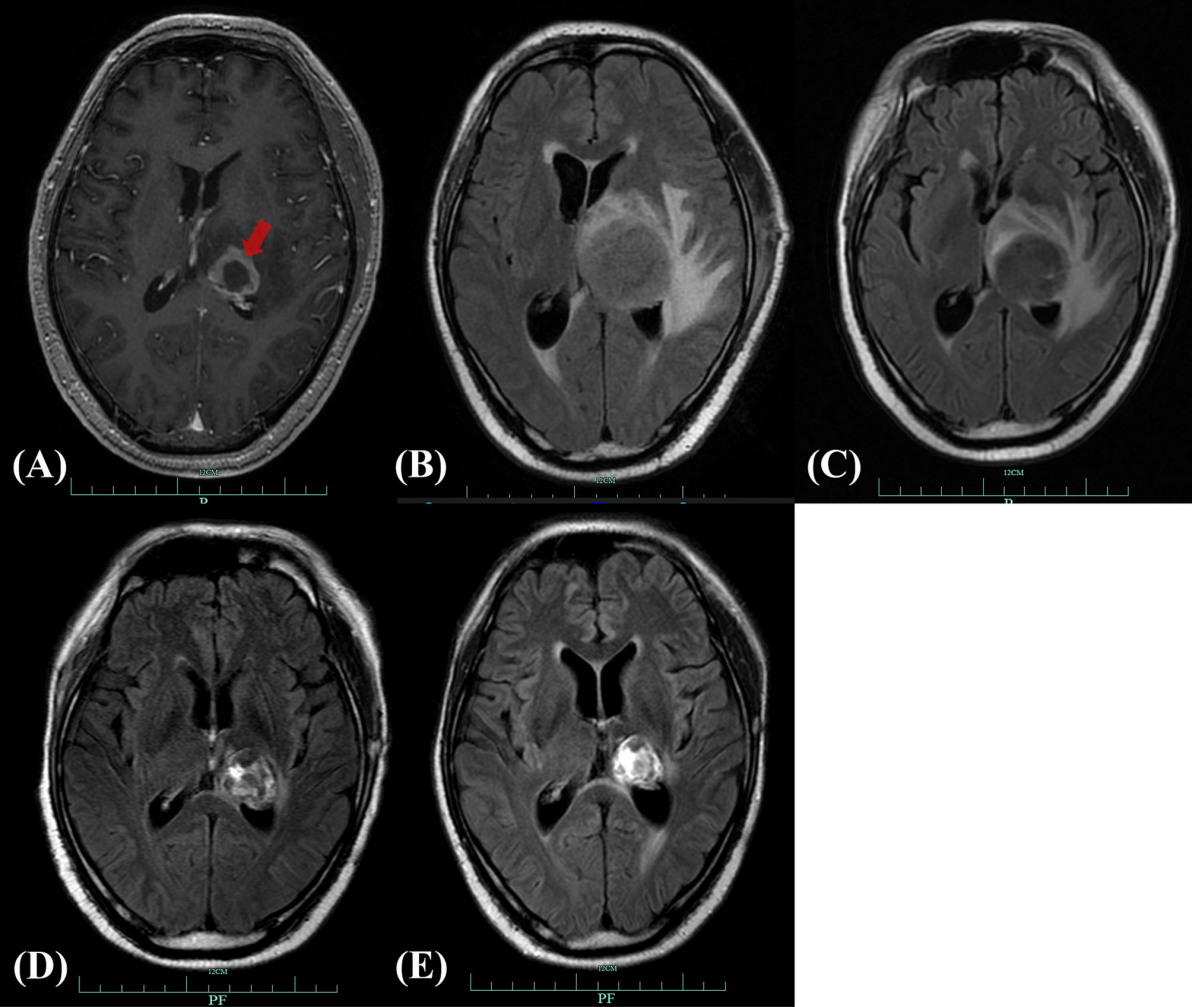
Brain MRI of CNS-PTLD. **(A)** Emergence of CNS-PTLD on day +225. MRI revealed a hypointensity lesion in the left thalamus with ring enhancement (red arrow) on contrast-enhanced T1-weighted imagining. **(B)** Day +255 (before treatment): the enlarged lesion surrounded by significant edema with the longest diameter of about 53mm (T2 Flair). **(C)** Day +280 (three weeks after the use of rituximab and MTX): reduction in the size of the lesion and edema with the longest diameter of 36.4mm (T2 Flair). **(D)** Day +363 (after whole-brain radiotherapy completed): further reduction in the size of the lesion with the longest diameter of 29mm (T2 Flair). **(E)** Day +477(three months after the start of zanubrutinib): the reduced lesion with the longest diameter of 24mm (T2 Flair). MRI, magnetic resonance imaging; CNS-PTLD, central nervous system post-transplant lymphoproliferative disorder.

**Figure 2:**
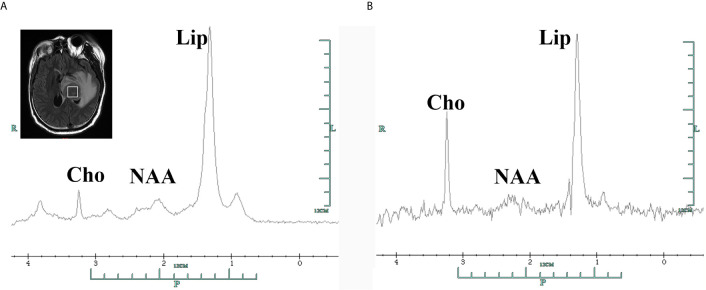
Single-voxel 1H-magnetic resonance spectroscopy of the tumor area in the left thalamus showing elevated Cho peak in 3.2 ppm and Lip peak in 1.3ppm with decreased NAA in 2.0 ppm, with corresponding short echo time spectra (**A**, TE=35ms) and long echo time spectra (**B**, TE=144ms). Cho, choline compounds; Lip, lipid; NAA, N-acetyl-aspartate.

**Figure 3:**
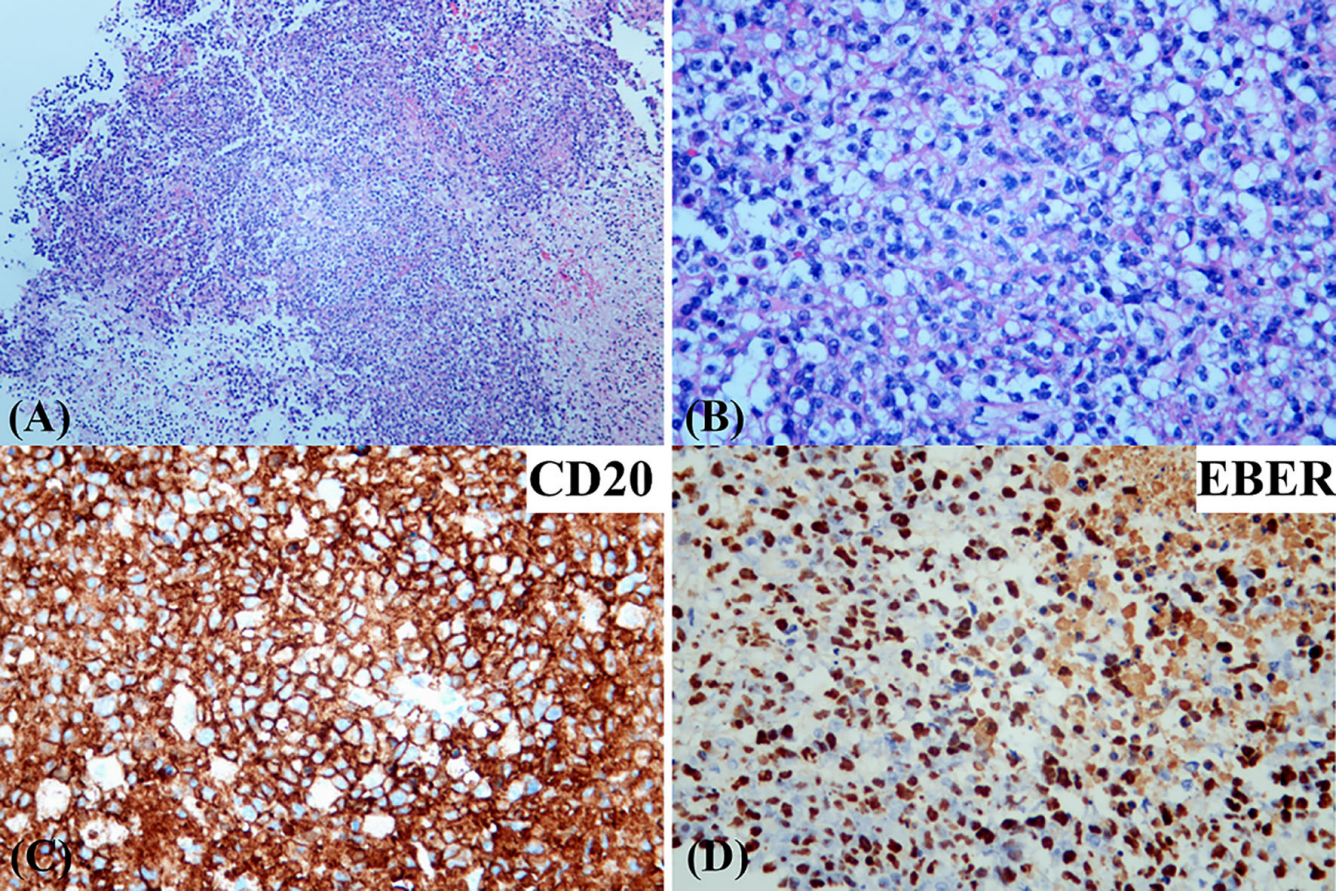
Photomicrographs of the brain biopsy demonstrating a monomorphic diffuse large B-cell lymphoma with EBV infection. [hematoxylin and eosin (H&E) stain; original magnification **(A)** ×50; **(B)** ×200]. Immunohistochemical stains showed that the infiltrating lymphocytes were positive for CD20 **(C)**. EBV-encoded RNA *in situ* hybridization was positive in the infiltrating cells **(D)**.

The patient presented with a recurring headache, accompanied by reducing right-sided power and binocular diplopia on day + 203 after HSCT, which underwent gradual exacerbation. He was readmitted to our center on day +223. Physical examination revealed grade IV muscle strength in the right limb and a positive Babinski sign on the right side. Brain MRI revealed a thalamic ring-enhancing lesion surrounded by edema on the left side (**Figure 1A**). CSF analyses demonstrated elevated cerebrospinal pressure (230 mmH_2_O) and elevated protein levels (1.280 g/L, reference <0.5 g/L). NGS of CSF was negative for bacterial, fungal, viral, or parasitic organisms. The serum Epstein-Barr virus (EBV)-DNA titer monitored twice a week ranged from 1.61×10^3^ to 4.0×10^4^copies/ml, with the treatment of ganciclovir. The distribution of T-cell subsets was examined: CD3+ T-cell count was 69 cells/μL, CD4+/CD8+ T cell was 0.57 and CD19+ B-cell count was 2 cells/μL, indicating that the patient was in a state of immunodeficiency. Systemic GVHD was not observed in the patient. Empirical treatment against Toxoplasma infection was implemented for 2 weeks but was ineffective. His clinical condition progressed on day +244 with grade II right-sided muscle strength, recurrent seizures, urinary incontinence, lethargy, memory loss, and transient consciousness disturbance. MRI was repeated, which revealed further enlargement of the lesion in the left thalamus, with a maximum diameter of approximately 53 mm (**Figure 1B**). We performed magnetic resonance spectroscopy (MRS), which depicted significant elevated lipid (Lip) and choline compounds (Cho) peaks, suggestive of lymphoma (**Figures 2A, B**). Brain biopsy, which was performed to confirm the diagnosis, identified monomorphic diffuse large B-cell lymphoma with EBV infection. The tumor cells stained positive for CD19, CD20, PAX-5, Ki-67 (approximately 65%), CD30, Bcl-2 (approximately 100%), MUM1, c-myc (approximately 25%), CD79a, and CD43 (**Figures 3A–C**). The results of EBV-encoded RNA *in situ* hybridization were positive (**Figure 3D**). Lung CT, abdominal B-ultrasound examination and bone marrow biopsy showed no evidence of systemic PTLD. Positron emission tomography-computed tomography (PET/CT) was not performed considering that the patient was critical with unstable conditions at that time. Based on these findings, the diagnosis of EBV-PTLD in central nervous system was made.

**Figure 4:**
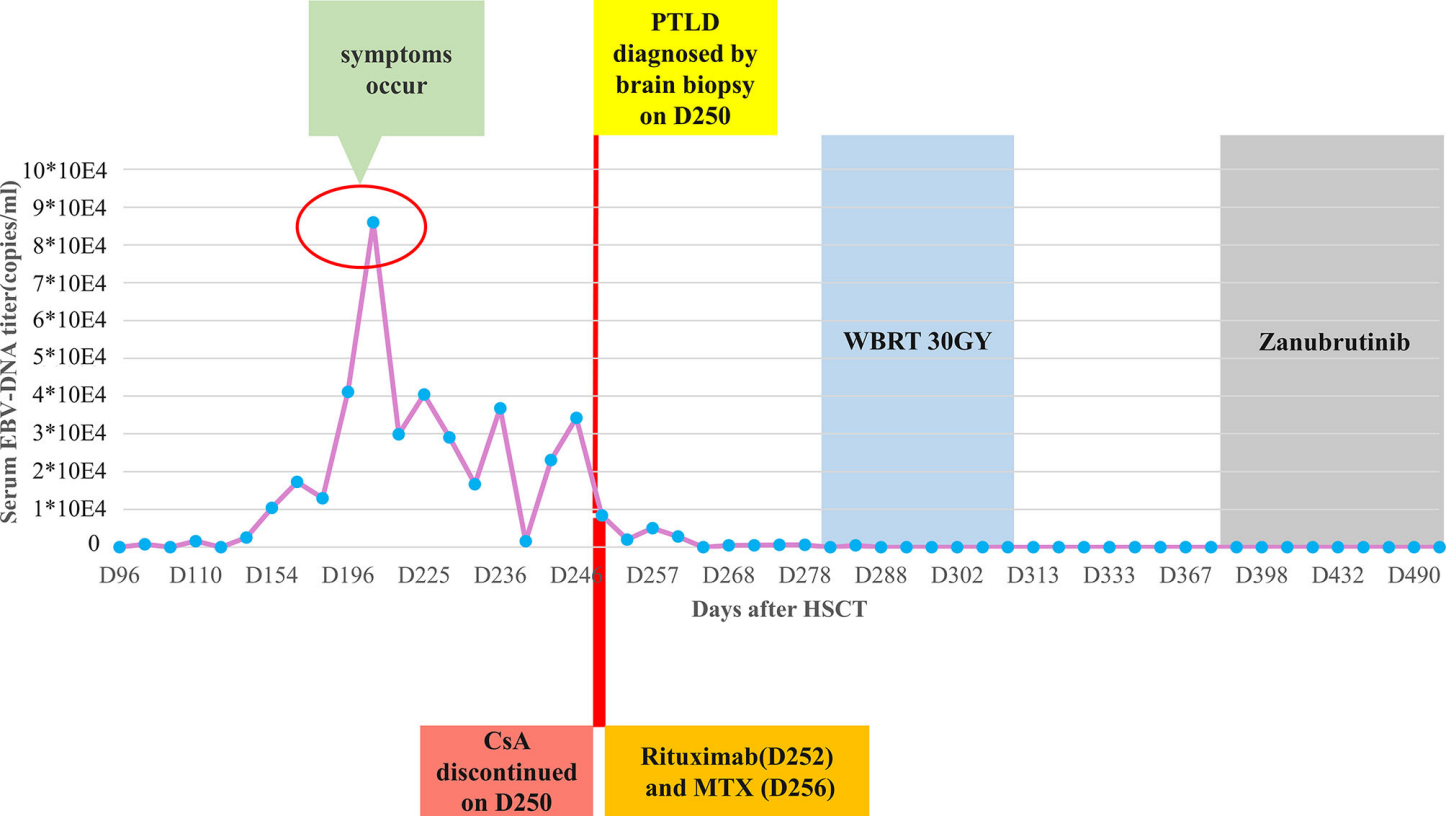
The clinical treatment process of posttransplant lymphoproliferative disorder (PTLD) after transplantation and the changes of serum EBV-DNA titers. HSCT, hematopoietic stem cell transplantation; CsA, cyclosporine; MTX, methotrexate; WBRT, whole-brain radiotherapy.

Thus, CsA administration (50mg daily) was discontinued at the diagnosis of PTLD on day +250. A single dose of rituximab 375 mg/m^2^ was administrated on day +252, followed by high-dose methotrexate (MTX, 6 g) on day +256. After MTX+ rituximab, his consciousness improved and the seizure disappeared, although he still had a headache with a numerical rating scale (NRS) score of 3. Brain MRI indicated a reduction in lesion size, with the longest diameter of 36mm (**Figure 1C**). Serum EBV-DNA load decreased to 2 log_10_ within three weeks after administration of chemotherapy. The response to MTX+rituximab was stable disease. During the treatment of MTX+rituximab, the WBC and platelet counts decreased to a minimum of 0.9×10^9^/L and 28×10^9^/L, respectively. The patient also developed pulmonary infection. Considering that the patient’s inability to tolerate chemotherapy, whole-brain radiotherapy (WBRT) was implemented on day + 280 (30 times, total dose of 30 Gy) for 47 days. During radiotherapy, the platelet and white blood cell (WBC) counts decreased to a minimum of 34 x 10^9^/L and 0.9 x 10^9^/L, respectively, but gradually recovered to normal. At the end of radiotherapy, his headaches were alleviated (NRS score=2) and the language impairment and dyskinesia also recovered gradually. Brain MRI showed the remained lesion with the longest diameter of 29mm (**Figure 1D**) and the serum EBV DNA load in the blood reduced to an undetectable level. After radiotherapy, the patient achieved partial response (PR) and his condition was stable, but there were still residual intracranial lesions. The patient refused further systemic chemotherapy. Considering the therapeutic activity of bruton tyrosine kinase (BTK) inhibitors for CNS lymphomas, the patient was administered oral zanubrutinib 80 mg daily (given the concurrent administration of posaconazole for preventing fungal infection) on day +382. He developed transient systemic migrating muscle soreness on the first day during zanubrutinib therapy, which was ameliorated 1 day after discontinuation of the drug. Oral administration of zanubrutinib was subsequently continued without any other side-effects. Blood counts were monitored regularly: the lowest WBC count was 2.9 x 109/L, and the hemoglobin and platelet counts were within the normal range. After starting zanubrutinib, His dizziness and headache had resolved, and the findings of neurological examination were normal. The serum EBV-DNA loads remained negative during the treatment of zanubrutinib. Follow-up brain MRI revealed that the lesion’s size decreased to 24 x 24 x 21 mm (**Figure 1E**) with a response of PR. Until the last follow-up in February 2021(day +516), he was alive without and clinical symptom and continued to take zanubrutinib. The treatment process and changes of serum EBV-DNA titer are summarized in **Figure 4**.

## Discussion

To date, there has been no definite guideline or consensus for the optimal treatment for CNS-PTLD after HSCT. The available therapeutic options include the withdrawal of immunosuppressive agents, high-dose MTX and cytarabine, WBRT and adoptive immunotherapy with EBV-specific cytotoxic T lymphocytes (2, 3). Although the patient achieved a PR with significant improvement of clinical symptoms after chemotherapy+ rituximab and WBRT, his headache remained, and MRI showed apparent residual lesions. Subsequent systemic chemotherapy was not considered because the patient refused chemotherapy. EBV-specific cytotoxic T lymphocytes(CTLs) derived from EBV-seropositive transplantation donors or the third party is effective in treat EBV- induced lymphoproliferative diseases through attacking EBV-infected cells, with durable response (4). Doubrovina et al. reviewed 19 EBV-PTLD patients who received EBV-specific CTL infusion after HSCT; in this study, 13 patients (85%) achieved CR and GVHD didn’t occur in any patients (5). However, EBV-specific CTLs was unavailable in our center.

BTK, which is a tyrosine-protein kinase, is critical to B-cell maturation and proliferation, has emerged as a significant therapeutic target for various B-cell malignancies (6, 7). The first-generation BTK inhibitor ibrutinib has shown promising results for CNS lymphoma (8, 9). A phase I clinical trial conducted by Grommes et al. reported that ibrutinib showed a 77% (10/13) clinical response in patients with relapsed or refractory CNS lymphoma, including CR and partial response in 5 patients each (9). Zanubrutinib (BGB-3111), a highly-specific, irreversible second generation BTK inhibitor developed in China, has greater selectivity and higher anti-tumor activity for BTK compared to ibrutinib. It shows more restricted off-target activity for a series of kinases, such as interleukin-2-induced kinases (ITK), Scr family kinases, and epidermal growth factor receptor (EGFR), thereby limiting the toxicity and side-effects (10). It has been approved for relapsed/refractory mantle cell lymphoma and chronic lymphocytic leukemia/small lymphocytic lymphoma. Moreover, its utility for the treatment of other B-cell malignancies is also being investigated worldwide. In our case, a further decrease in the size of lesions was observed 3 months after the use of zanubrutinib, suggesting its efficacy in treating CNS PTLD. The drug is well-tolerated, and no obvious hematological toxicity or infection was observed during the period of treatment. The patient should take zanubrutinib consistently until treatment failure or the occurrence of unacceptable toxicities. To the best of our knowledge, this was the first report to describe treatment of CNS-PTLD with zanubrutinib. However, it is not sure whether the response of zanubrutinib for PTLD is durable or further improved. Longer follow-up is needed to evaluate its effect.

## Conclusion

Our case shows that the novel BTK inhibitor zanubrutinib exhibit specific activity for CNS lymphomas. It provides a potential therapeutic option for CNS-PTLD when other attempted measures are judged to be ineffective or inappropriate.

## Data Availability Statement

The original contributions presented in the study are included in the article/**Supplementary Material**. Further inquiries can be directed to the corresponding author.

## Ethics Statement

The studies involving human participants were reviewed and approved by the ethics review committee of the First Affiliated Hospital of Zhejiang University School of Medicine. The patients/participants provided their written informed consent to participate in this study.

## Author Contributions

T-TY wrote the manuscript. W-HC, Y-MZ, H-RF and HH contributed to the patient’s medical care. J-MS edited and approved the manuscript. All authors contributed to the article and approved the submitted version.

## Funding

This work was funded by the National Natural Science Foundation of China (Grant no. 82070179).

## Conflict of Interest

The authors declare that the research was conducted in the absence of any commercial or financial relationships that could be construed as a potential conflict of interest.

## Supplementary Material

Supplementary Figure 1 Brain Magnetic resonance imaging of cerebral toxoplasmosis. Multiple hyperintense enhancing lesions in bilateral cerebral hemispheres and cerebellar before treatment (on day +94 after transplantation) were observed **(A–C)**. Two months after initiation of anti-toxoplasma therapy (on day +153 after transplantation), lesions had nearly disappeared **(D, E)**. Red arrow indicated enhancing lesions on contrast-enhanced T1-weighted imaging.
