# Supplementary figures and images for: Zanubrutinib Treatment of Central Nervous System Posttransplant Lymphoproliferative Disorder After Allogeneic Hematopoietic Stem Cell Transplantation: A Case Report
Source: Front Oncol. 2021 Apr 29;11:672052. doi: 10.3389/fonc.2021.672052 (PMC8117139; doi:10.3389/fonc.2021.672052)

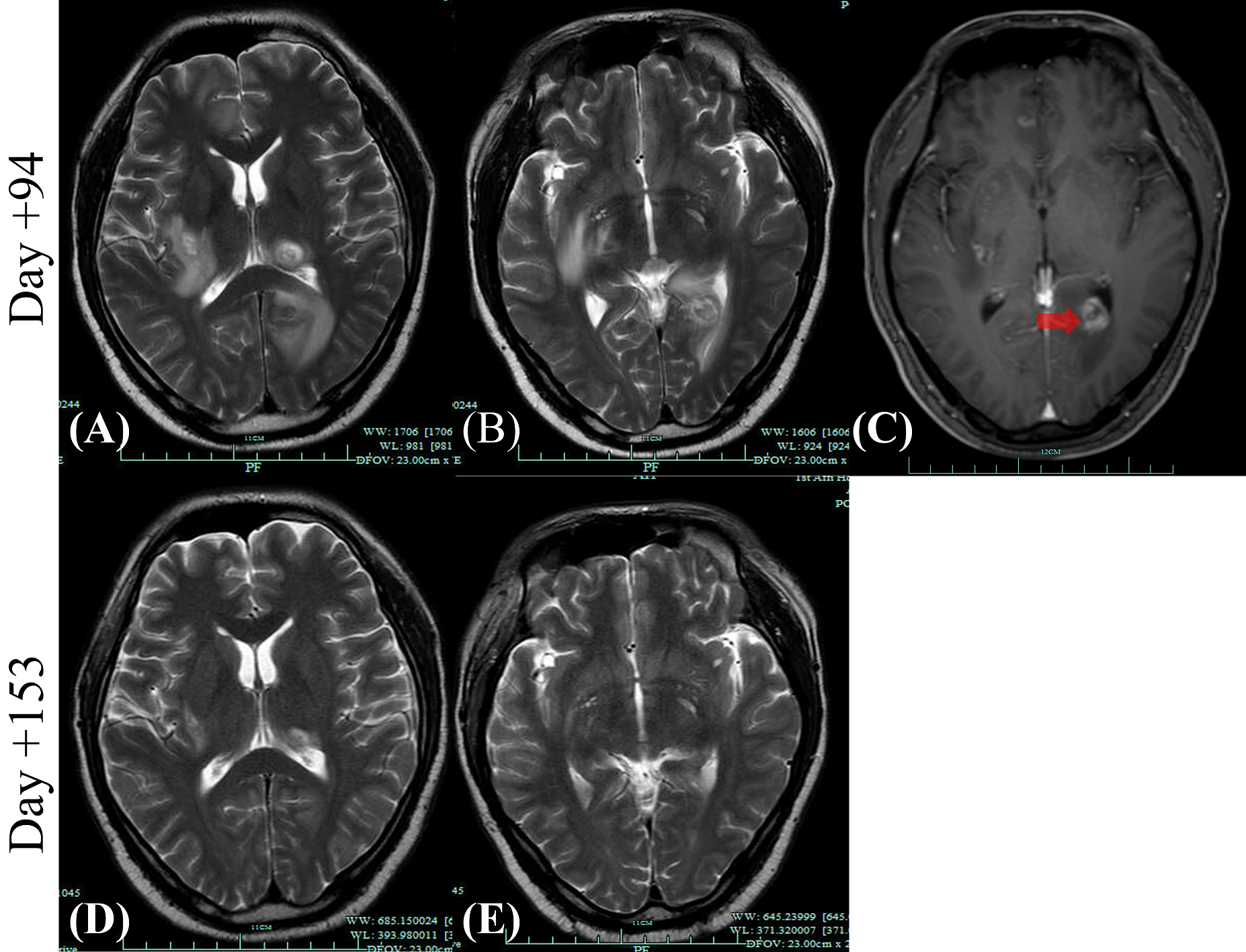

Supplement: Supplementary Figure 1 — Brain Magnetic resonance imaging of cerebral toxoplasmosis. Multiple hyperintense enhancing lesions in bilateral cerebral hemispheres and cerebellar before treatment (on day +94 after transplantation) were observed (A–C). Two months after initiation of anti-toxoplasma therapy (on day +153 after transplantation), lesions had nearly disappeared (D, E). Red arrow indicated enhancing lesions on contrast-enhanced T1-weighted imaging. [file Image_1.tif]
